# Inferior frontal gyrus preserves working memory and emotional learning under conditions of impaired noradrenergic signaling

**DOI:** 10.3389/fnbeh.2013.00197

**Published:** 2013-12-17

**Authors:** Benjamin Becker, Lucas Androsch, Ralph T. Jahn, Therese Alich, Nadine Striepens, Sebastian Markett, Wolfgang Maier, René Hurlemann

**Affiliations:** ^1^Department of Psychiatry and Psychotherapy, University of BonnBonn, Germany; ^2^Department of Psychology, University of BonnBonn, Germany; ^3^German Center for Neurodegenerative Diseases (DZNE)Bonn, Germany

**Keywords:** compensation, fMRI, noradrenaline, propranolol, working memory, emotional learning, inferior frontal cortex, cognitive control

## Abstract

Compensation has been widely applied to explain neuroimaging findings in neuropsychiatric patients. Functional compensation is often invoked when patients display equal performance and increased neural activity in comparison to healthy controls. According to the compensatory hypothesis increased activity allows the brain to maintain cognitive performance despite underlying neuropathological changes. Due to methodological and pathology-related issues, however, the functional relevance of the increased activity and the specific brain regions involved in the compensatory response remain unclear. An experimental approach that allows a transient induction of compensatory responses in the healthy brain could help to overcome these issues. To this end we used the non-selective beta-blocker propranolol to pharmacologically induce sub-optimal noradrenergic signaling in healthy participants. In two independent functional MRI (fMRI) experiments participants received either placebo or propranolol before they underwent a cognitive challenge (Experiment 1: working memory; Experiment 2: emotional learning: Pavlovian fear conditioning). In Experiment 1 propranolol had no effects on working memory performance, but evoked stronger activity in the left inferior frontal gyrus (IFG). In Experiment 2 propranolol produced no effects on emotional memory formation, but evoked stronger activity in the right IFG. The present finding that sub-optimal beta-adrenergic signaling did not disrupt performance and concomitantly increased IFG activity is consistent with, and extends, current perspectives on functional compensation. Together, our findings suggest that under conditions of impaired noradrenergic signaling, heightened activity in brain regions located within the cognitive control network, particularly the IFG, may reflect compensatory operations subserving the maintenance of behavioral performance.

## Introduction

The theoretical construct “neural compensation” is widely applied to explain functional neuroimaging results in neuropsychiatric patients. Functional compensation is often invoked when populations with presumed pathological or age-related brain changes display intact cognitive performance and concomitantly enhanced neural activity in comparison to healthy controls (Maruishi et al., 2007; Schwindt and Black, 2009; Erk et al., 2011; Koester et al., 2013). According to current concepts of neural compensation, increased activity is thought to reflect compensatory operations which subserve the maintenance of cognitive functions despite neuropathological changes (Park and Reuter-Lorenz, 2009; Grady, 2012). Neural compensation has been implicated in various neuropsychiatric disorders, including Alzheimer's disease (AD), brain damage, affective illness, or drug addiction (Erk et al., 2011; Etkin and Schatzberg, 2011; Becker et al., 2012; Koester et al., 2013). Most frequently compensatory mechanisms have been observed in AD patients. Extensive degeneration of the locus coeruleus-norephineprine (LC-NE) system is universal in this disease [for an overview see (Chalermpalanupap et al., 2013)] and altered LC-NE functioning is among the earliest pathologies of AD (Mann et al., 1980; Haglund et al., 2006), which even can be detected during early prodromal stages (Grudzien et al., 2007; Braak and Del Tredici, 2011). LC-NE degeneration has been closely linked to cognitive decline in AD (Weinshenker, 2008) and animal models of AD have revealed compelling evidence that elevated NE signaling could ameliorate cognitive deficits in AD. Despite the continued attention the compensatory concept has received in the context of neuropathological diseases, particularly AD, important issues still remain unsolved. Depending on the cognitive function and the specific clinical population under investigation, increased activity in several brain regions has been attributed to compensatory mechanisms. Recent meta-analytic data from AD patients suggest that prefrontal regions, particularly ventrolateral and dorsolateral subdivisions of the prefrontal cortex, are critically involved in compensation (Schwindt and Black, 2009). However, several pathology-related issues, such as confounding effects of task performance (Gould et al., 2005, 2006), cortical atrophy (Johnson et al., 2000) and lower baseline activity in patients (Lustig et al., 2003; Greicius et al., 2004) might have influenced the results of these analyses. It is therefore mandatory to study neural compensation in the absence of pathological brain changes. Given the association between decreased LC-NE signaling in AD, one experimental strategy to transiently model compensatory responses in the healthy brain might be the pharmacological inhibition of NE signaling.

Biosynthesis of the neurotransmitter norepinephrine (NE, noradrenaline) depends on the nutritional availability of its amino acid precursor tyrosine (Fernstrom and Fernstrom, 1994), and NE homeostasis is crucial for a variety of brain regions receiving noradrenergic innervation, including the prefrontal cortex and amygdala (Robbins and Arnsten, 2009; Gamo and Arnsten, 2011). Consequently, disruptions of NE signaling in prefrontal cortex have been linked to the etiology of several neuropsychiatric disorders such as major depression, attention-deficit/hyperactivity disorder (ADHD), and AD (West et al., 2009; Arnsten and Pliszka, 2011; Robertson, 2013). Traditionally, the cortical NE system has been associated with basic functions such as arousal and sleep-wake cycle (Jouvet, 1969; Aston-Jones et al., 1999), but current perspectives suggest that NE has a key role in signal detection and behavioral control (Sara, 2009), thus optimizing task performance under impaired conditions (Aston-Jones and Cohen, 2005). Specifically, NE is thought to exert an inverted-U shaped dose effect on prefrontal cortex functioning, such that intermediate signals are required for regular performance and either too little (e.g., through depletion) or too much (e.g., due to uncontrollable stress) NE activity impairs performance (Greene et al., 2009). NE signaling in prefrontal cortex has been implicated in fundamental cognitive functions (Berridge and Waterhouse, 2003; Robbins and Arnsten, 2009), including working memory and emotional learning (Chamberlain et al., 2006), both of which are highly preserved across species and crucial for survival. Substantial evidence for a central role of NE in these memory functions comes from challenge studies in which the α_2_-adrenergic agonist clonidine or the non-selective β_1–3_-adrenergic antagonist propranolol modulated working memory and emotional memory formation (Davis et al., 1979; Cahill et al., 1994; Franowicz and Arnsten, 1999; Li et al., 1999; Strange and Dolan, 2004; Hurlemann, 2008; Hurlemann et al., 2010; Tully and Bolshakov, 2010). Given this intimate functional relationship, and in view of evolutionary perspectives suggesting that survival stress and malnutrition are the rule rather than the exception in driving adaptation, the question emerges if there is evidence for a neural compensation of transient fluctuations in NE signaling. To address this question, we administered, in two separate double-blind, randomized, placebo-controlled functional MRI (fMRI) experiments, a 40-mg single oral dose of propranolol to healthy volunteers in order to disrupt noradrenergic signaling via β-noradrenergic receptors. Specifically, the rationale was to induce a transient state of sub-optimal functioning of NE-dependent cognitive operations in the domains of (i) working memory and (ii) emotional memory formation. The latter was assessed using a classical Pavlovian fear conditioning paradigm. We hypothesized that our experimental intervention would elicit compensatory neural responses in the absence of behavioral performance deficits.

## Materials and methods

### Experimental design and procedures

**Figure 1:**
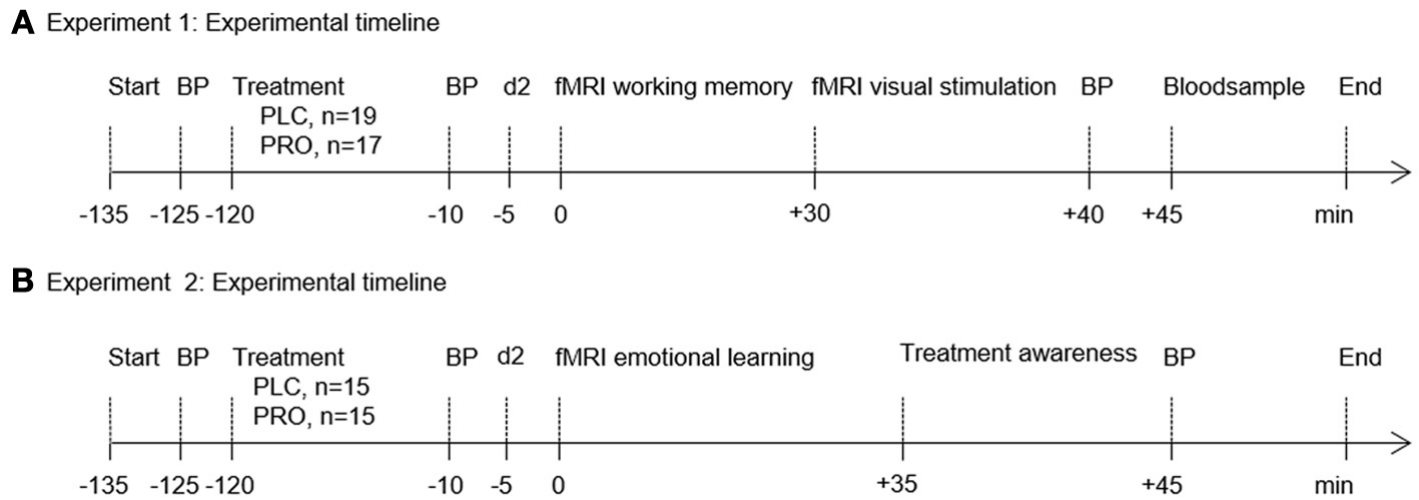
**Experimental timelines and fMRI paradigms**. Experimental timeline of Experiment 1 **(A)** and Experiment 2 **(B)**. In both experiments volunteers were administered either placebo (PLC) or propranolol (PRO) 120 min before testing. Blood pressure (BP) was measured at the time of verum/placebo administration as well as immediately before and after the scanning session. To control for confounding effects of propranolol on attention all subjects completed the d2 test (“Aufmerksamkeits- und Belastungstest d2”) (Brickenkamp, 1995) immediately before the scanning session. In Experiment 1 venous blood samples were drawn for propranolol plasma level analysis. In Experiment 2 participants were administered a questionnaire to assess treatment awareness. Abbreviations: PLC, placebo; PRO, propranolol; BP, blood pressure.

The rationale of this randomized, double-blind, placebo-controlled trial was to challenge compensatory mechanisms that enable the human brain to preserve cognitive functioning. In independent experiments, two fundamental cognitive functions vital for survival (Experiment 1, working memory; Experiment 2, emotional learning) were challenged in young, healthy participants. In both experiments the centrally and peripherally acting non-selective β-adrenergic antagonist propranolol (PRO) was used to pharmacologically induce transient suboptimal noradrenergic functioning in young, healthy participants with a presumably high compensatory potential. In view of the pharmacokinetic profile of PRO (time to peak plasma concentration, 1–3 h; elimination half-life, 3–4 h), participants received either PRO or lactose placebo (PLC) 120 min before the start of each experiment. We administered a 40-mg single oral dose of PRO in accordance with our previous studies, which documented behavioral and neural effects in healthy subjects (Hurlemann et al., 2005, 2010; Onur et al., 2009). Blood pressure was measured at the time of PRO/PLC administration as well as immediately before and after the scanning session. Paired *t*-test comparisons revealed a (trend to) significant decrease in systolic blood pressure from baseline to pre fMRI measurement (Experiment 1 *p* = 0.11; mean, s.e.m. at baseline, 121.42, 1.80; mean s.e.m. at pre-fMRI, 118.89, 1.61; Experiment 2 *p* = 0.07; mean, s.e.m. at baseline, 122.30, 2.55; mean s.e.m. at pre-fMRI, 118.76, 3.20) and from baseline to post fMRI measurement (Experiment 1 *p* = 0.05; mean s.e.m. at post-fMRI, 116.47, 2.34; Experiment 2 *p* = 0.03; mean s.e.m. at post-fMRI, 119.07, 2.69) in the PRO group. No significant change was observed in the PLC group (all *p* > 0.51). Interindividual variation in the degree of first-pass metabolism contributes to the differences in PRO plasma levels after oral administration of equivalent doses (Wood et al., 1978). Therefore, in Experiment 1 venous blood samples were collected immediately after scanning to determine the PRO plasma level in each subject. PRO concentrations in serum specimens were quantified in the Medical Laboratory Bremen (http://www.mlhb.de) using an LC-MS/MS approach. In brief, sera were deproteinized with acetonitrile/methanol after addition of methylrisperidone as the internal standard. Samples were then centrifuged and supernatants were diluted with mobile phase (acetonitrile/acetic acid). After separation from matrix components by HPLC (Agilent 1100C; Agilent Technologies, Santa Clara, CA), selected ion fragments produced via electrospray ionization were detected and quantified in a Sciex API4000 (Applied Biosystems, Carlsbad, CA) triple-quadrupole mass spectrometer. Assays were calibrated with spiked pool sera, the detection limit (signal/noise ratio = 3) was 1 μg/L, and the imprecision (coefficient of variation) within series (*n* = 10) amounted to 8.2%. Mean plasma concentrations were 24.3 ± 9.52 μg/L. To account for potential effects of medication awareness participants were asked to rate whether they received PRO or PLC at the end of Experiment 2. For a schematic synopsis and experimental timelines of the experiments see Figure 1 (Experiment 1, Figure 1A; Experiment 2, Figure 1B).

### Participants

For both experiments healthy, young right-handed volunteers were recruited by advertisement and provided written informed consent. All volunteers were free of current or past medical (including respiratory or allergic illness), neurological, or psychiatric disorders as assessed by medical history and a structured clinical interview for DSM-IV and ICD-10 psychiatric disorders (Mini-International Neuropsychiatric Interview; M.I.N.I) (Sheehan et al., 1998). Volunteers were naïve to prescription-strength psychoactive medication [including propranolol for the treatment of “exam nerves” (Brewer, 1972)] and had not taken any over-the-counter psychoactive medication within the past 4 weeks. Tobacco smokers and volunteers with known contraindications for MRI scanning were excluded from participation. To control for pre-treatment differences in cognitive performance, all volunteers were administered a comprehensive neuropsychological test battery. Screening included the RAVLT (Rey Auditory Verbal Learning Test) (Rey, 1941; Helmstaedter et al., 2001) to assess verbal learning skills, the DST (digit-span test) derived from the revised Wechsler adult intelligence scale (Wechsler, 1997) to assess working memory, the LPS 4 (“Leistungspruefsystem Subtest 4”) (Horn, 1983) to assess non-verbal reasoning IQ, the MWT-B (“Mehrfach-Wortschatz-Intelligenztest Teil B”) (Lehrl, 1978) to assess verbal IQ based on lexical decisions, and the trail-making test (TMT) part A and B (Raitan, 1958) to assess visual attention and task-switching. Handedness was determined by the Edinburgh handedness inventory (EHI) (Oldfield, 1971). The study had full ethical approval and was carried out in compliance with the latest revision of the Declaration of Helsinki.

A total of 36 participants (18 females, mean age, 24.4 years; age range 21–32 years) were enrolled in Experiment 1 and received either a 40-mg single oral dose of PRO (*n* = 17; 7 females) or PLC (*n* = 19; 11 females) before the they were administered the spatial working memory task and the visual stimulation paradigm. The treatment groups were of comparable age, gender distribution, education, and pre-treatment neuropsychological performance (all *p*-values > 0.05) (Table 1). For Experiment 2 30 participants were recruited (14 females, 25.1 years; age range 21–31 years) and received either a 40-mg single oral dose of PRO (*n* = 15; 6 females) or PLC (*n* = 15; 8 females) before they were administered the emotional learning paradigm. Due to technical failures during data acquisition of the aversive learning paradigm the data from 5 participants had to be excluded from further analysis (PRO, *n* = 2; PLC, *n* = 3). The treatment groups (after exclusion of the 5 participants, PRO, *n* = 13, 5 females; PLC, *n* = 12, 6 females) were comparable in terms of age, gender distribution, education, and pre-treatment neuropsychological performance (Table 1). To control for confounding effects of PRO on attention, subjects in both experiments completed the d2 test (“Aufmerksamkeits- und Belastungstest d2”) (Brickenkamp, 1995) immediately before scanning. Participants were instructed to maintain their regular sleep and wake schedule and to abstain from caffeine and alcohol intake on the day before scanning.

**Table 1:** **Two-sample *t*-tests showed no significant pretreatment differences in demographic and neuropsychological characteristics between the placebo- and propranolol-treated groups (displayed are mean and *SD* for both groups)**.

**Experiment 1**	**Placebo (***n*** = **19**)**	**Propranolol (***n*** = **17**)**
Age, years	24.41 (±3.26)	25.76 (±3.08)
Education, years	16.50 (±2.13)	16.29 (±1.51)
Gender distribution (f:m)	10:7	8:11
RAVLT		
Trial 1–5^a^	59.31 (±7.42)	58.47 (±7.42)
Trial 5^b^	13.15 (±2.05)	13.41 (±1.62)
Trial 6 Retention^c^	12.73 (±2.40)	12.58 (±2.26)
Trial 7 Delayed Recall^d^	12.63 (±2.26)	13.05 (±2.26)
MWT-B^e^	30.89 (±2.20)	30.06 (±2.21)
TMT-A^f^	22.87 (±8.90)	24.81 (±6.32)
TMT-B^g^	55.62 (±14.47)	67.31 (±21.95)
Digit-span, forward^h^	8.36 (±1.64)	8.17 (± 2.27)
Digit-span, backwards^i^	7.05 (±1.50)	7.76 (±2.35)
**Experiment 2**	**Placebo (***n*** = **12**)**	**Propranolol (***n*** = **13**)**
Age, years	24.43 (±2.50)	24.25 (±3.23)
Education, years	16.50 (±2.12)	17.46 (±1.75)
Gender distribution (f:m)	6:6	8:5
RAVLT		
Trial 1–5^a^	57.80 (±8.29)	60.23 (±9.05)
Trial 5^b^	13.41 (±1.67)	13.38 (±2.02)
Trial 6 Retention^c^	12.50 (±2.31)	12.15 (±2.67)
Trial 7 Delayed Recall^d^	12.83 (±2.32)	12.69 (±2.46)
MWT-B^e^	30.00 (±3.90)	28.93 (±4.09)
TMT-A^f^	25.75 (±6.90)	26.81 (±8.32)
TMT-B^g^	64.25 (±18.47)	60.31 (±16.95)
Digit-span, forward^h^	7.83 (±2.37)	7.53 (± 1.54)
Digit-span, backwards^i^	7.58 (±2.15)	7.69 (±2.56)

RAVLT, Rey Auditory Verbal Learning.

a Learning performance across five trials.

b Supraspan.

c Susceptibility to interference.

d Delayed recall after 30 min; MWT-B, Mehrfachwahl-Wortschatz-Intelligenz-Test.

e Number of correct responses, TMT-A, TMT-B, Trail-Making Test A, B.

f, g Processing time inseconds; DST, digit-span forward and backward test.

h, i Number of digits correctly recalled.

### Experimental paradigms

#### Experiment 1—spatial working memory paradigm

We administered an adapted version of an event-related visuospatial delayed matching-to-sample working memory task (McNab and Klingberg, 2008; Markett et al., 2010). Participants had to remember a variable number of positions during a delay and to compare a probe location with the sample stimulus locations maintained in working memory. After a 500-ms fixation period, a circular grid divided into 16 sections was presented for 1000 ms as the sample stimulus. In the grid one, three, or five red squares appeared in random positions with the constraint that no more than two squares were located in adjacent locations. After a retention interval of 2500 ms the grid was presented again for 2000 ms with one position cued (probe). Participants had to indicate by button press whether or not the location of the probe matched the position of one of the red squares of the sample stimulus (left index finger: no match, right index finger: match). Working memory load was manipulated by displaying 1, 3, or 5 red squares during the sample period. Participants were instructed to respond as fast as possible while avoiding errors. Forty trials per load condition (one, three, and five positions), distributed across two runs, were presented in randomized order in an event-related design. Trial duration was 6000 ms and the mean duration of the jittered intertrial interval was 3720 ms (2500–4500 ms, in 500 ms steps). Each run had a mean duration of ~7.5 min; resulting in a total duration of 15 min. In total, 152 dynamic scans were recorded per run, resulting in 254 dynamic scans for the entire experiment.

#### Experiment 1—visual stimulation paradigm

We used a 10 × 10 checkerboard with green-to-red switches at a frequency of 8 per second over a block with duration of 16 s. Six blocks of checkerboard stimulation alternated with six blocks of rest, at which a blank screen was presented.

#### Experiment 2—aversive learning paradigm

Pavlovian fear conditioning is highly conserved across species, thus providing a powerful model to study compensatory mechanisms during emotional memory formation.

***Stimuli***. The unconditioned stimulus (UCS) consisted of brief electrical shocks of 2 ms duration. The electrical shock stimuli were delivered via a SHK1 aversive shock stimulator (Contact Precision Instruments, Cambridge, MA) coupled with a notebook computer. Current was passed from the generator to the subject via two Ag/AgCl electrodes filled with electrolyte gel on the subject's left (non-dominant) dorsal lower arm. Before acquisition, shock intensity levels were set manually for each individual by delivering gradually more intense shocks (0–5 mA range) until the subject reported the shock was “highly annoying yet not painful.” The two conditioned stimuli (CS+, which was paired with the UCS in 75% contingency that coterminated with the CS+, and CS−, which was never paired with the UCS) consisted of one male and one female face from the Ekman series (Ekman and Friesen, 1971) whose hair was removed. Mildly (20%) angry faces were chosen on previous studies showing successful conditioning with mildly angry faces (Critchley et al., 2002; Kalisch et al., 2006). The two same faces were used for all subjects, CS+ and CS− assignment to the male and female face was balanced across treatment and gender of the participants. During the conditioning procedure the skin conductance responses (SCRs) were sampled simultaneously with MR scans. SCRs were acquired at a sampling rate of 1000 Hz from Ag/AgCl electrodes filled with isotonic electrolyte gel on the middle and ring finger of the left (non-dominant) hand by means of a SC5 24-bit Skin Conductance System (Contact Precision Instruments, Cambridge, MA).

***Instruction***. Subjects were told the intention to study face processing under stress and were debriefed at the end of the study. To ensure attentional processing of the stimuli the subjects performed an unspeeded gender decision task with the index finger (male) and thumb (female) of their dominant (right) hand.

***Conditioning procedure***. Subjects were first habituated to the CSs by presenting each CS five times before the conditioning procedure. Throughout the acquisition procedure the CS+ and CS− were presented 38 times for 6000 ms in a randomized order (restriction: no more than two consecutive presentations of one CS). The CS+ was followed by shock in 75% contingency. CSs were separated by a variable interstimulus interval (ISI) ranging from 8 to 11 s, during which subject viewed a central fixation cross (low-level baseline). Following the acquisition phase, contingency was measured via a questionnaire asking participants whether they had noticed any relationship between the shock and the gender of the face.

***MRI data acquisition***. For MRI acquisition a 1.5 Tesla Siemens Avanto MRI system (Siemens, Erlangen, Germany) was used. Task-related fMRI data were acquired using a T2^*^-weighted echoplanar EPI sequence (imaging parameters: *TR* = 3000 ms, *TE* = 50 ms, matrix size: 64 × 64, pixel size: 3 × 3 × 3 mm, slice thickness = 3.0 mm, distance factor = 10%, FoV = 210, flip angle = 90°, 35 axial slices). In addition, high-resolution anatomical images were acquired on the same scanner using a T1-weighted 3D MPRAGE sequence (imaging parameters: *TR* = 1660 ms, *TE* = 3.09, matrix size 256 × 256, pixel size 1 × 1 mm^2^, slice thickness = 1.0 mm, FoV = 256, flip angle = 15°, 160 sagittal slices).

### fMRI data analysis

#### Preprocessing

Task-related fMRI data were preprocessed and analyzed using SPM8 software (Wellcome Trust Centre for Neuroimaging, London, United Kingdom; http://www.fil.ion.ucl.ac.uk/spm) implemented in Matlab 7 (The MathWorks Inc., Natick, MA). The first five volumes of each functional time series were discarded to allow for T1 equilibration. Images were corrected for head movement between scans by an affine registration (Ashburner and Friston, 2003). For realignment, a two-pass procedure was used, by which images were initially realigned to the first image of the time-series and subsequently re-realigned to the mean of all images. For spatial normalization, the mean EPI image of each subject was normalized to the current Montreal Neurological Institute (MNI) template (Evans et al., 1992; Holmes et al., 1998) using the unified segmentation function implemented in SPM8. This algorithm combines image registration, tissue classification, and bias correction within the same generative model. All images were hereby transformed into standard stereotaxic space and resampled at 3 × 3 × 3 mm voxel size. The normalized images were spatially smoothed using an 8-mm FWHM Gaussian kernel. Raw time series were detrended by the application of a high-pass filter (cut-off period, 128 s).

#### Working memory paradigm

Separate onset regressors for the three levels of WM load were modeled by a stick function convolved with a hemodynamic response function (Friston et al., 1994). The movement parameters were included as confounds. Specific effects were assessed by contrasting the load 5 and load 3 conditions with the load 1 condition, which served as a baseline due to its negligible engagement of working memory resources. Effects of treatment were analyzed by directly comparing the PRO and the PLC treatment group by means of *t*-tests entering the contrasts “load 3 > load 1” and “load 5 > load 1.”

#### Visual stimulation paradigm

The visual stimulation was modeled by a boxcar function convolved with a canonical hemodynamic response function. The design matrix comprised contrasts of alternating intervals of visual stimulation and rest, the time derivative, and the six head movement parameters as confounds. Specific effects were assessed by contrasting the visual stimulation condition with the rest condition. Effects of treatment were analyzed by directly comparing the PRO- and the PLC-treated groups using a two-sample *t*-test.

#### Emotional learning paradigm

Separate onset regressors for the CS+, CS−, and the UCS were modeled by a stick function convolved with a hemodynamic response function (Friston et al., 1994). The movement parameters were included as confounds. Specific effects of the conditioning procedure in the entire sample were assessed by contrasting the CS+ with the CS−. We used structurally defined regions of interest to enhance the statistical power in regions that have consistently been implicated in fear-conditioning [systematic review in (Sehlmeyer et al., 2009)]: amygdala, insula and anterior cingulate cortex. Effects of treatment were analyzed by directly comparing the PRO and the PLC treatment group by means a *t*-test entering the contrast “CS+ > CS−.”

#### ROI definition

Previous literature indicates consistently greater, and presumably compensatory, activity in ventrolateral PFC (vlPFC) and dorsolateral PFC (dlPFC) regions among patient populations (Maruishi et al., 2007; Schwindt and Black, 2009; Smith et al., 2010). Based on these results, the analysis of effects of suboptimal noradrenergic functioning was focused on these regions. Regions-of-interest (ROIs) were anatomically defined using the WFU Pickatlas (Version 3.0), which provides a method for generating ROI masks based on the Talairach Daemon database (Tzourio-Mazoyer et al., 2002; Maldjian et al., 2003, 2004). The inferior frontal gyrus (IFG) served as the ROI for the vlPFC and Brodmann area (BA) 9 served as the dlPFC ROI. ROI-based two-sample *t*-tests were computed with a threshold of *p* < 0.05 and Family-Wise Error (FWE) corrected for multiple comparisons.

### Skin conductance data analysis

Skin conductance data were downsampled to 100 Hz and visually inspected for artifacts. Data from the first 5 subjects had to be discarded due to severe artifacts in the MRI and SCR data (PRO, *n* = 2; PLC, *n* = 3). After installing custom-made filters between the MRI control room and the scanner room artifacts could be controlled for the subsequently scanned subjects. All subsequent analyses of the emotional learning data were therefore performed using the data acquired from 25 subjects (PRO, *n* = 13, 5 females; PLC, *n* = 12, 6 females). In line with previous investigations an SCR was defined as the maximum of the SCR signal in the time window of 4.8 s after CS onset minus an SCR baseline value (the mean of the SCR in the second before the onset of the SCR) (Buchel et al., 1998; Kalisch et al., 2006). To account for interindividual differences in physiological reactivity, SCR data were z-transformed (Buchel et al., 1998). Significance of SCR difference between CS− and CS+ was assessed using a Two-Way repeated measures ANOVA with treatment (PLC vs. PRO) as between-subject factor and stimulus type (CS+ vs. CS−) as within-subject factor. Based on the previous literature that allowed a directed hypotheses for the effects of conditioning (CS+ > CS−), a one-tailed threshold of *p* = 0.05 was used for the SCR data.

## Results

### Experiment 1

#### Behavioral data (working memory performance)

**Figure 2:**
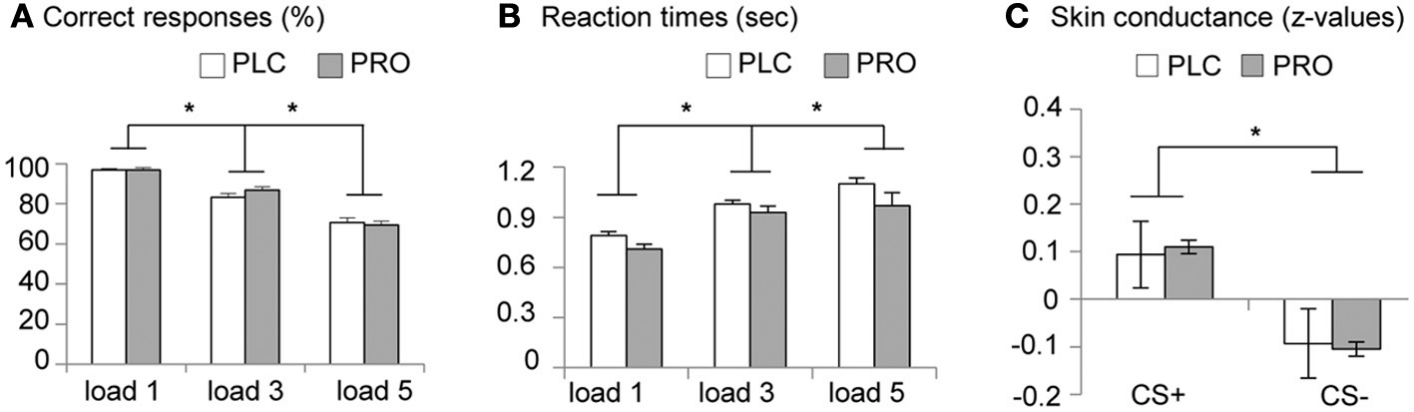
**Behavioral data**. Placebo- and propranolol-treated subjects did not differ in **(A)** working memory accuracy and **(B)** response latencies (^*^p < 0.05, two-tailed). **(C)** Larger SCRs during CS+ vs. CS− throughout the conditioning procedure suggests successful conditioning. Z scores were used instead of raw values (microSiemens) to account for interindividual differences. (^*^p < 0.05, one-tailed). Displayed are group-mean values and corresponding standard errors of the mean (s.e.m.). Abbreviations: PLC, placebo; PRO, propranolol.

Analysis of the attentional performance data acquired immediately before scanning revealed no significant performance differences between the PLC- and PRO-treated subjects [*t*_(34)_ = 0.39, *p* = 0.69]. This indicates that effects of a β-adrenergic blockade on working memory are not confounded by reduced attentional capacities. Analysis of working memory accuracy (percent correct responses), using a Two-Way repeated measures ANOVA with treatment (PLC vs. PRO) as between-subject factor and working memory load (load 1, load 3, and load 5) as within-subject factor, revealed a significant main effect of working memory load [*F*_(2, 33)_ = 200.42, *p* < 0.001]. *Post-hoc* analyses using paired *t-tests* revealed that subjects displayed a gradual decrease in percent correct responses from load 1 (96.8 ± 4.3%) over load 2 (84.9 ± 8.4%) to load 3 (70.1 ± 9.3%) with significant performance differences between load 3 and load 2 [*t*_(35)_ = 11.9, *p* < 0.001] and load 2 and load 1 [*t*_(35)_ = 9.1, *p* < 0.001]. This confirms an increase in working memory demands across the three load conditions. Neither the main effect of treatment [*F*_(1, 34)_ = 0.123, *p* > 0.05] nor the treatment × load interaction effect [*F*_(2, 33)_ = 0.158, *p* > 0.05] reached statistical significance, indicating that PRO had neither a general nor load-specific effect on working memory accuracy (Figure 2A). The analysis of the response latencies for correct responses using a Two-Way repeated ANOVA revealed a significant main effect of working memory load [*F*_(2, 33)_ = 58.41, *p* > 0.001], thereby confirming increasing task difficulty across the three load conditions. In addition, there was a trend to significant main effect of treatment [*F*_(1, 33)_ = 3.46, *p* > 0.05], indicating shorter response latencies in the PRO-treated subjects. However, the treatment × load interaction effect failed to reach statistical significance [*F*_(2, 33)_ = 3.46, *p* > 0.05], indicating that there was no load-specific effect of PRO (Table 2; Figure 2B).

**Table 2:** **Behavioral results from the working memory task**.

	**Placebo (***n*** = **19**)**	**Propranolol (***n*** = **17**)**
**LOAD 1**		
Response accuracy (percent correct)	96.85 (0.70)	96.75 (1.31)
Response latencies (in ms)	796 (25)	711 (29)
**LOAD 2**		
Response accuracy (percent correct)	83.17 (2.02)	86.72 (1.89)
Response latencies (in ms)	981 (23)	930 (38)
**LOAD 3**		
Response accuracy (percent correct)	70.68 (2.38)	69.38 (1.99)
Response latencies (in ms)	1115 (36)	974 (78)

Mean values and s.e.m. are displayed.

#### fMRI data—working memory

**Figure 3:**
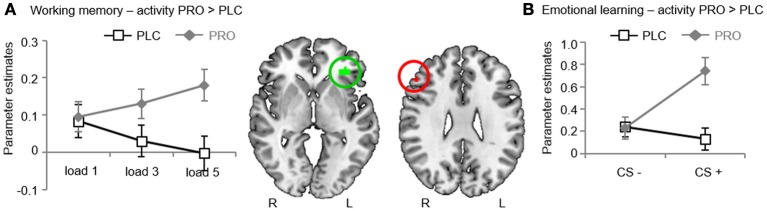
**fMRI results**. **(A)** Experiment 1. During high working memory load propranolol-treated subjects showed stronger activity in the left inferior frontal gyrus (IFG). Plotting the individual extracted parameter estimates for the left IFG revealed that activity in this region increased with working memory load after propranolol, yet not placebo treatment. **(B)** Experiment 2. During emotional memory acquisition propranolol-treated subjects showed stronger activity in the right IFG. Plotting the individual extracted parameter estimates for the right IFG revealed this effect was driven by selective effects of propranolol on the CS+. Abbreviations: PLC, placebo; PRO, propranolol; IFG, inferior frontal gyrus; CS+, conditioned stimulus.

In line with previous fMRI studies using spatial working memory paradigms (Wager and Smith, 2003; Rottschy et al., 2012) we found increased activity in the fronto-parietal working memory networks for both working memory contrasts (“load 3 > load 1”; “load 5 > load 1”) (see also Table 3 for the main effect of WM load). Anatomically restricted comparisons between the treatment groups revealed significantly increased activity in the pars triangularis subdivision of the left IFG of the PRO-treated subjects for the contrast “load 5 > load 1” [*t*_(34)_ = 6.87; *p* < 0.05, FWE-corrected; maximum *t*-value located in MNI-space at *x* = −33, *y* = 35, *z* = −2] (Figure 3A). There were no significant differences in the opposite direction or for the contrast “load 3 > load 1.” An exploratory whole-brain analysis revealed that the effect in the left IFG remained significant on the whole-brain level. In addition, this analysis revealed no other regions with significant between-group differences, indicating regional-specific effects. For illustration purposes the individual parameter estimates were extracted from a sphere (6 mm radius) centered at the coordinates of the maximum *t*-value (Figure 3A). To explore associations between WM performance and IFG activity bivariate correlations (Pearson) between the behavioral performance measures (percent correct responses, response latencies) and the extracted IFG parameter, estimates were computed within each group. However, this analysis revealed no significant results (results are given in Table 4). Taken together, results from Experiment 1 indicate that PRO had no effects on working memory performance but concomitantly increased activity in the pars triangularis subdivision of the left IFG under conditions of high working memory load.

**Table 3:** **Significant main effect of working memory load (“load 3 + load 5 > load 1”) in the entire sample (*n* = 36) thresholded at *p* > 0.05 (FWE-corrected)**.

**Region**	**Number of voxels**	**MNI-coordinates (*x*/*y*/*z*)**	**Maximum *t*-value**
Insula R	279	33/23/−2	11.80
Insula L	446	−33/23/−5	11.55
Middle occipital gyrus R	481	39/−76/34	10.09
Postcentral gyrus L	637	−51/−34/55	9.81
Inferior frontal gyrus R	337	48/35/25	9.43
Middle frontal gyrus L	102	−33/53/16	9.21
Superior frontal gyrus	235	0/29/37	9.11
Thalamus L	87	−12/−4/10	8.87
Precuneus L	390	−6/−70/52	8.77
Culmen R	32	3/−52/−8	8.64
Inferior temporal gyrus R	22	51/−52/−5	7.78
Rolandic Operculum R	51	48/−19/19	7.09

**Table 4:** **Bivariate correlations between the extracted parameter estimates from the left inferior frontal gyrus and working memory performance**.

	**Placebo (***n*** = **19**)**	**Propranolol (***n*** = **17**)**
**LOAD 1**		
IFG activity—response accuracy	−0.25 (*p* = 0.30)	0.14 (*p* = 0.60)
IFG activity—response latencies	0.12 (*p* = 0.64)	−0.08 (*p* = 0.78)
**LOAD 2**		
IFG activity—response accuracy	0.17 (*p* = 0.33)	−0.02 (*p* = 0.95)
IFG activity—response latencies	−0.14 (*p* = 0.57)	0.05 (*p* = 0.85)
**LOAD 3**		
IFG activity—response accuracy	0.38 (*p* = 0.11)	0.18 (*p* = 0.48)
IFG activity—response latencies	−0.15 (*p* = 0.55)	−0.04 (*p* = 0.90)

#### fMRI data—visual stimulation

Analysis of the whole sample indicated that the checkerboard stimulation paradigm elicited robust neural responses in the bilateral visual cortex. Direct comparison of the PRO- and PLC-treated groups revealed no significant differential activations, arguing against non-specific treatment effects possibly resulting from globally decreased noradrenergic receptor activity or homogenous changes in cerebral hemodynamics.

### Experiment 2

#### Behavioral data (treatment and contingency awareness)

In the post-scan questionnaire 5 (PLC, *n* = 3; PRO, *n* = 2) participants answered that they could not rate whether they received PRO or PLC. From the remaining participants (PLC, *n* = 9; PRO, *n* = 11) 4 in the PLC and 5 in the PRO group correctly identified their treatment. A *Chi*^2^-test confirmed that the correct identification in both groups was not greater than chance (*Chi*^2^_1_ = 0.20, *p* > 0.05). Of the 25 subjects 20 (PLC, *n* = 10; PRO, *n* = 10) reported awareness of the CS+—UCS contingency in the post-scan questionnaire. All participants performed the gender decision task with a high degree of accuracy (percent correct responses: PLC, CS+, 98.7 ± 2.1%; CS−, 98.5 ± 2.0%; PRO, CS+, 98.6 ± 2.0%; CS−, 99.1 ± 1.7%).

#### Skin conductance data (emotional learning)

Visual inspection of the SCR data indicated that 8 participants (PLC, *n* = 3; PRO, *n* = 5) did not show any apparent SCR to the UCS and therefore were excluded from further analysis. Mean standardized SCRs from the remaining subjects were subjected to a repeated measures ANOVA with the between subject factor treatment (PLC vs. PRO) and the within subject factor CS type (CS+ vs. CS−). Results from an analysis including CS+—UCS contingency aware participants (PLC, *n* = 8; PRO, *n* = 6) confirmed successful conditioning by a significant main effect of CS type [*F*_(1, 12)_ = 5.47, *p* < 0.05, one-tailed; *post hoc* paired *t*-test, *t*_(13)_ = 2.44, *p* < 0.05, one-tailed; mean *z*-value, s.e.m.; CS+, 0.099, 0.04; CS−, −0.099, 0.04]. However, neither the main effect of treatment [*F*_(1, 12)_ = 0.13 *p* > 0.05] nor the treatment × CS type interaction effect reached statistical significance [*F*_(2, 12)_ = 0.02, *p* > 0.05]. (the corresponding data are presented in Table 5, Figure 2C). Together these results suggest that beta-noradrenergic blockade did not interfere with contingency awareness or the acquisition of conditioned autonomic responses during the conditioning procedure.

**Table 5:** **SCR responses from the emotional learning task**.

	**Placebo (***n*** = **8**)**	**Propranolol (***n*** = **6**)**
**SCR RESPONSE (*z*-VALUES)**		
CS+	0.09 (0.07)	0.11 (0.01)
CS−	−0.09 (0.07)	−0.11 (0.02)
CS+ >	0.19 (0.15)	−0.21 (0.03)
CS−		

Mean z-standardized SCR responses and s.e.m. are displayed.

#### fMRI data—emotional learning

In line with previous fMRI studies using aversive conditioning procedures (Sehlmeyer et al., 2009) we found that the CS+ resulted in stronger BOLD responses than the CS− in the bilateral anterior cingulate (ACC) [left ACC, *t*_(24)_ = 4.32, MNI coordinates *x* = −4, *y* = 32, *z* = 22; right ACC, *t*_(24)_ = 4.46, MNI coordinates *x* = 2, *y* = 22, *z* = 26; *p* < 0.05; FWE-corrected] and the bilateral insula (INS) [left INS, *t*_(24)_ = 4.41, MNI coordinates *x* = −30, *y* = 20, *z* = 8; right INS, *t*_(24)_ = 5.17, MNI coordinates *x* = 36, *y* = 20, *z* = 8; *p* < 0.05; FWE-corrected] in the entire sample (see Table 6). Anatomically restricted comparisons between the CS+-UCS contingency aware participants in the treatment groups (PLC, *n* = 10; PRO, *n* = 10) revealed significantly increased activity in the pars triangularis subdivision of the right IFG of the PRO-treated subjects for the contrast “CS+ > CS−” [*t*_(18)_ = 6.42; *p* < 0.05, FWE-corrected; maximum *t*-value located in MNI-space at *x* = 50, *y* = 32, *z* = 28] (Figure 3B). Findings remained significant when all participants were included [PRO, *n* = 13; PLC, *n* = 12; *t*_(23)_ = 5.34; *p* < 0.05, FWE-corrected; maximum *t*-value located in MNI-space at *x* = 50, *y* = 32, *z* = 28]. An exploratory whole-brain analysis for the entire sample revealed no other regions with significant between-group differences. However, the effect in the right IFG also failed to reach statistical significance on the whole-brain level. Subsequently extracted individual parameter estimates from a 6 mm radius sphere centered at the coordinates of the maximum *t*-value from the between group comparison, revealed, that PRO selectively increased activity for the CS+ (Figure 3B, group differences for both tasks are summarized in Table 7).

**Table 6:** **Significant main effect of stimulus type (“CS+ > CS−”) in the entire sample (*n* = 25) thresholded at *p* > 0.05 (FWE-corrected for the size of the ROI)**.

**Region of Interest (ROI)**	**Maximum *t*-value**	**MNI-coordinates (*x*/*y*/*z*)**
Insula R	5.17	−36/20/8
Insula L	4.41	30/20/8
Anterior cingulate cortex R	4.46	2/22/26
Anterior cingulate cortex L	5.39	−4/32/22

**Table 7:** **Significant between-group differences from both experiments thresholded at *p* > 0.05 (FWE-corrected for the size of the ROI)**.

**Paradigm**	**Region of Interest (ROI)**	**MNI-coordinates (*x*/*y*/*z*)**	**Maximum *t*-value**
Working memory PRO > PLC (“load 5 > load 1”)	Inferior frontal gyrus L	−33/35/−2	6.87
Emotional memory PRO > PLC (“CS+ > CS−”)	Inferior frontal gyrus R	50/32/28	5.34

## Discussion

The hypothesis of neural compensation has been widely used to explain a pattern of intact cognitive performance and concomitantly increased activity in neuropsychiatric populations. In the present randomized, double-blind, placebo-controlled pharmacological fMRI study the β-receptor antagonist propranolol was used to model conditions of (transient) suboptimal noradrenergic functioning in healthy young subjects performing working memory (Experiment 1) and emotional learning (Experiment 2) tasks. Consistent with our initial hypothesis, β-receptor blockade with propranolol did not influence, or interfere with, working memory or emotional learning performance in healthy young subjects. Indeed, both cognitive functions showed a high resistance against transient variation in NE signaling. Of note, across both experiments the lack of performance decrements was accompanied by increased activity in the IFG during cognitive challenge.

Before an in depth discussion of the present findings limitations of the present experimental approach need to be considered. In the present study we used pharmacological fMRI as an innovative approach to elicit neural compensation in the healthy brain. However, one constraint of the fMRI methodology is it does not allow causal brain-behavior relations to be drawn. Conclusions regarding the functional relevance of the increased activity observed during the present study thus must remain preliminary. One possible way to address this issue in future studies could be the use of different doses of propranolol. Using increasing doses of propranolol should ultimately lead to compensatory failure and apparent behavioral impairments. Conversely, propranolol in higher doses has been shown to affect downstream cognitive domains. Particularly confounding effects of β-receptor blockade on attention (Aston-Jones et al., 1999; De Martino et al., 2008) might certainly influence, and interfere with processes in higher-order cognitive domains. In addition, increasing doses of propranolol produce significant decreases in heart rate and blood pressure (Campbell et al., 2008; de Rover et al., 2012). In fMRI experiments these cardiac-related effects can lead to global susceptibility effects caused by pulsatile motion of the brain as well as local blood pressure-related changes in the BOLD signal (Hagberg et al., 2008). Another possible way to examine the functional relevance of the increased PFC activity could be the use of non-invasive brain stimulation techniques. For instance, repetitive transcranial magnetic stimulation (rTMS) has been used successfully to disturb cognitive performance by discharging neurons located in the cortex (Walsh and Pascual-Leone, 2003) and is thus ideally suited to disrupt putative compensatory activity in the IFG. Given that the increased activity is engaged in the functional compensation its disruption by rTMS should lead to a failure of compensation and unmask performance impairments. Still, our data provide evidence for the functional relevance of the observed response increases in the IFG. First, propranolol selectively increased activity and connectivity under conditions of cognitive challenge (high working memory load, CS+) but had no effect during the control conditions (attentional control condition, CS−). Second, in accordance with a putative compensatory increase, activity and connectivity of the prefrontal cortex regions increased with working memory load. Consistent with the compensatory hypothesis, these findings indicate that neural effects of suboptimal NE signaling were specifically associated to, and increased with, cognitive load.

The observed lack of behavioral effects of a 40-mg single dose of propranolol on working memory and emotional memory seems contradictory to previous findings. However, behavioral studies in healthy volunteers that used comparable single-dose application schemes yielded inconsistent effects of propranolol on working memory performance. Muller and colleagues reported decreased working memory performance in healthy young volunteers following a 25-mg single-dose application of propranolol (Muller et al., 2005). While, others found no (Alexander et al., 2007) or even beneficial effects of a 40-mg propranolol dose on working memory performance (Campbell et al., 2008). Differences might be explained by the inverted U shaped effect NE has on cognitive performance. Different cognitive functions have distinct levels of optimal noradrenergic activity (Robbins, 2000) and consequently show different sensitivities for beta-adrenergic blockade. Negative effects on working memory performance were specifically observed during the manipulation of information maintained in working memory (Muller et al., 2005). In contrast, the working memory task administered in the present study required short-term maintenance only. Unexpectedly, we observed a trend for shorter response latencies in the propranolol-treated subjects. This effect might reflect the beneficial effects of beta-adrenergic antagonists under conditions of psychological stress (Ramos et al., 2005; Alexander et al., 2007). Effects of propranolol on emotional memory encoding in healthy volunteers have been extensively studied. The majority of studies used emotional stimuli (e.g., emotional slides or words) and found reduced recall for emotionally salient material (overview by Chamberlain and Robbins, 2013). Effects of propranolol on the acquisition of fear conditioning in humans have been studied by Grillon et al. (2004) who, in line with the present findings, did not observe strong effects on cued fear conditioning. Compared with simple maintenance or fear conditioning, the manipulation of working memory content or the encoding of emotional slides involve more complex cognitive operations, which might render these behavioral paradigms more susceptible to the effects of beta-adrenergic blockade. In addition, previous studies observing no effects on simple short-term maintenance or the acquisition of cued fear responses (Grillon et al., 2004; Alexander et al., 2007) assessed only behavioral effects, thus potential compensatory mechanisms on the neural level cannot be excluded.

Across both experiments propranolol consistently increased activity in the pars triangularis subdivision of the IFG (albeit on contralateral sides) under cognitive challenge. Consistent with several functional imaging studies in neuropsychiatric patients our current data thus suggest a crucial role of the IFG in maintaining cognitive performance under conditions of suboptimal NE signaling. Meta-analytic data from fMRI studies on episodic memory in AD patients revealed consistently increased IFG activity among these patients (Schwindt and Black, 2009). Support for the functional relevance of IFG activity increases in AD patients comes from two studies in which IFG activity predicted better cognitive status (Diamond et al., 2007) and retrieval success (Grady et al., 2003) among AD patients. Importantly, studies in cognitively intact adults at genetic risk for AD have provided further support for a specific involvement of the IFG in compensatory mechanisms (Wishart et al., 2006; Filbey et al., 2010). Moreover, our current results extend these previous findings in neuropsychiatric populations and suggest that the IFG is not only involved in the adaptation to slow progressing neuropathological changes, but also supports compensation of transient fluctuations in NE signaling. Considerable evidence suggests that the IFG is critically implicated in the flexible adjustment of cognitive control, particularly through cognitive inhibition (Rubia et al., 2003; Aron et al., 2004). Cognitive control refers to the ability to perform task-relevant cognitive operations in the face of interference (Badre and Wagner, 2007; Levy and Wagner, 2011). More recently the IFG has been implicated in a superordinate domain-general cognitive control network that supports different executive functions, including flexibility, working memory, and inhibition (Cole and Schneider, 2007; Niendam et al., 2012). Therefore, the present pattern of increased IFG activity during cognitive challenge may indicate that propranolol-treated subjects achieved normal performance through increasing cognitive control demands. This interpretation would be in accord with the widely accepted role of NE signaling in optimizing behavioral performance and in improving signal-to-noise ratio (Arnsten et al., 1996; Aston-Jones and Cohen, 2005).

Although propranolol produced increased IFG activity across both tasks, the observed increases showed a task-specific laterality. Differences in laterality might be related to the cognitive domain that was challenged. Cognitive control refers to the ability to perform task-relevant cognitive operations in the face of interference and considerable evidence has associated the bilateral IFG with this function (Badre and Wagner, 2007; Levy and Wagner, 2011). Some, however, not all, studies reported that the left and right IFG may sub-serve specific sub-processes of cognitive control. The left IFG in particular has been associated with interference reduction during the maintenance phase of working memory processing. This region has shown increased activity under conditions of high interference in the Sternberg working memory task as compared to a low-interference condition of the same task (Jonides et al., 1998). In another study, left IFG responses were correlated with the ability to resolve interference efficiently during working memory processes (Bunge et al., 2001). In contrast, the contralateral (right) IFG has been suggested to respond to currently relevant and salient stimuli to guide attention (Hampshire et al., 2010). In line with this proposed function right IFG activity has repeatedly been observed when pre-learnt objects are detected (Linden et al., 1999; Hampshire et al., 2007). Greater IFG activity in propranolol-treated subjects in both tasks may therefore reflect greater effort to remain focused on the salient stimuli and filter out distracting information. This interpretation would be in accord with the widely accepted role of LC-NE signaling in optimizing behavioral performance and in improving signal-to-noise ratio (Arnsten et al., 1996; Aston-Jones and Cohen, 2005).

A potential constraint of the present study is the lack of propranolol-induced decreased activity often observed in AD patients and other neuropsychiatric populations. However, studies with cognitively intact adults at high risk for AD display increased prefrontal activity during working memory without any regions showing decreased activity (Wishart et al., 2006; Bokde et al., 2010; Lancaster et al., 2011; Chen et al., 2013).

In conclusion, the present finding that suboptimal β-adrenergic signaling did not disrupt performance in the context of concomitantly increased IFG activity is consistent with the pattern widely explained by the compensatory hypothesis. Our findings thus suggest that (i) increasing activity may represent a common brain mechanism to maintain performance and (ii) that regions within the cognitive control network, particularly the IFG, are involved in compensatory mechanisms.

## Author contributions

Benjamin Becker and René Hurlemann designed the study, interpreted the data and wrote the paper. Nadine Striepens, Lucas Androsch, Ralph T. Jahn, and Therese Alich performed the experiments and analyzed behavioral data. Sebastian Markett and Wolfgang Maier gave technical support and conceptual advice. All authors discussed the results and implications and commented on the manuscript.

### Conflict of interest statement

The authors declare that the research was conducted in the absence of any commercial or financial relationships that could be construed as a potential conflict of interest.
